# Screening of Secreted Proteins of *Sporisorium reilianum* f. sp. z*eae* for Cell Death Suppression in *Nicotiana benthamiana*

**DOI:** 10.3389/fpls.2020.00095

**Published:** 2020-02-19

**Authors:** Deiziane Dutra, Nisha Agrawal, Hassan Ghareeb, Jan Schirawski

**Affiliations:** ^1^ Microbial Genetics, Institute of Applied Microbiology, RWTH Aachen University, Aachen, Germany; ^2^ Genetics, Matthias-Schleiden-Institute, Friedrich-Schiller-University Jena, Jena, Germany; ^3^ Plant Biotechnology, National Research Centre, Cairo, Egypt; ^4^ Molecular Biology of Plant-Microbe Interactions, Albrecht-von-Haller Institute of Plant Sciences, Schwann-Schleiden Research Center, Georg-August-University Göttingen, Göttingen, Germany

**Keywords:** *Sporisorium reilianum*, plant pathogen, hypersensitive response, effector proteins, *Nicotiana benthamiana*, agroinfiltration, INF1 elicitin, cell death suppression

## Abstract

*Sporisorium reilianum* f. sp. *zeae* (SRZ) is a biotrophic fungus causing head smut in maize. Maize infection with SRZ leads to very little cell death suggesting the presence of cell-death suppressinpg effectors. Several hundred effector proteins have been predicted based on genome annotation, genome comparison, and bioinformatic analysis. For only very few of these effectors, an involvement in virulence has been shown. In this work, we started to test a considerable subset of these predicted effector proteins for a possible function in suppressing cell death. We generated an expression library of 62 proteins of SRZ under the control of a strong constitutive plant promoter for delivery into plant cells *via Agrobacterium tumefaciens*-mediated transient transformation. Potential apoplastic effectors with high cysteine content were cloned with signal peptide while potential intracellular effectors were also cloned without signal peptide to ensure proper localization after expression in plant cells. After infiltration of *Nicotiana benthamiana* leaves, infiltration sites were evaluated for apparent signs of hypersensitive cell death in absence or presence of the elicitin INF1 of *Phytophthora infestans*. None of the tested candidates was able to induce cell death, and most were unable to suppress INF1-induced cell death. However, the screen revealed one predicted cytoplasmic effector (sr16441) of SRZ that was able to reliably suppress INF1-induced cell death when transiently expressed in *N. benthamiana* lacking its predicted secretion signal peptide. This way, we discovered a putative function for one new effector of SRZ.

## Introduction

The phytopathogenic biotrophic basidiomycete *Sporisorium reilianum* f. sp. *zeae* (SRZ) is the causative agent of head smut disease of maize. The disease can cause great damage and leads to complete harvest loss of affected individual plants. In nature, the disease is transmitted by soil-borne diploid teliospores. Under favorable environmental conditions, teliospores germinate, undergo meiosis, and generate haploid sporidia of different mating types (Hanna, 1929; Halisky, 1963; Martinez et al., 2002). Prior to plant infection, compatible haploid sporidia form conjugation hyphae that grow toward each other and fuse at their tips (Schirawski et al., 2005). After mating, the fungus then grows as dikaryotic hyphae that penetrate and colonize the plant initially without causing severe symptoms (Martinez et al., 1999; Prom et al., 2011; Poloni and Schirawski, 2016). Symptoms become evident only at the flowering time when spore formation and phyllody occur in the inflorescences (Wilson and Frederiksen, 1970; Martinez et al., 1999; Ghareeb et al., 2011; Poloni and Schirawski, 2016).

For host plant colonization, pathogens have to overcome several lines of plant defense. Plant defense mechanisms allow perception of pathogen attack and activation of pre- and postinvasion defense responses to minimize damages imposed by destructive invaders (Dangle and Jones, 2001). Plant pathogens acquired the ability to defeat plant immunity responses, resulting in a co-evolutionary arms race for resistance or susceptibility. The first line of defense of the plant's innate immune system is provided by pattern recognition receptors (PRRs) that recognize conserved microbial- or pathogen-associated molecular patterns (MAMPs or PAMPs) and trigger the so-called PAMP-triggered immunity (PTI) response (Jones and Dangl, 2006; Coll et al., 2011). Pathogens can overcome PTI-based defenses by expressing specific effectors that suppress PTI and lead to effector-triggered-susceptibility (ETS) (Coll et al., 2011; Asai and Shirasu, 2015).

Effector proteins secreted by successful plant pathogens modulate and reprogram the defense systems of the host. Two types of secreted effector proteins are known: Apoplastic effectors that are targeted to the plant extracellular space, and cytoplasmic effectors that are delivered inside the plant cell and target different subcellular compartments (Bos et al., 2006; Kamoun, 2006; Asai and Shirasu, 2015). Effectors may be recognized by plant disease resistance (R) proteins, which may result in hypersensitive response (HR), a form of programmed cell death (PCD). Host cell death does not always have a negative impact on the plant. Targeted destruction of specific plant cells can be a powerful mechanism of defense against biotrophic plant pathogens that rely on living host cells to colonize and complete their infection cycles (Lam, 2004; Reape et al., 2007). The infection success of biotrophic pathogens is therefore determined by the ability of the pathogen to suppress the induction of plant defense responses leading to programmed cell death. Suppression of PCD through secretion of specific effectors delivered into host cells has been shown for many different systems. The effector protein AvrPiz-t secreted by *Magnaporthe oryzae* suppresses the mouse BAX-induced programmed cell death in *Nicotiana benthamiana* leaves (Li et al., 2009), while MoHEG13 antagonizes cell death induced by *M. oryzae* Necrosis-and ethylene-inducing-protein-1 (Nep1)-like proteins in *N. benthamiana* (Mogga et al., 2016). The Avr3a effector of *Phytophthora infestans* interacts with the potato U-box E3 ubiquitin ligase CMPG1 and stabilizes it to suppress Infestin1 (INF1)-mediated cell death (Bos et al., 2010; Derevnina et al., 2016). INF1 is a *P. infestans* elicitin inducing HR cell death. Elicitins are highly conserved extracellular proteins secreted by phytopathogenic microorganisms that have features of pathogen-associated molecular patterns (PAMPs) and trigger defenses in a variety of plant species (Du et al., 2015; Derevnina et al., 2016). Expression of *P. infestans* INF1 is largely used in *N. benthamiana* to screen for effectors that function as immunosuppressants. The effector AVR3a-KI, a *P. infestans* host-translocated (cytoplasmic) effector, suppresses the HR cell death triggered by INF1 (Bos et al., 2006).

To successfully infect maize, *S. reilianum* f. sp. *zeae* is predicted to secrete hundreds of effector proteins (Schirawski et al., 2010; Schuster et al., 2018; Schweizer et al., 2018; Ghareeb et al., 2019) that facilitate modulation of plant innate immunity and colonization of the host tissue (Ghareeb et al., 2019), supposedly by suppressing plant innate immune responses. For only very few effectors, an involvement in virulence has been shown (Ghareeb et al., 2015; Schweizer et al., 2018; Ghareeb et al., 2019). Since plant penetration is followed by a long phase of fungal proliferation within the plant tissue but without prominent disease symptoms, some effectors likely function in suppressing cell death.

In the present study, we aimed to assign a function to more effector proteins by identifying effectors that could suppress cell death. We selected a set of bioinformatically predicted small secreted proteins from SRZ, created an expression library of 62 constructs and expressed them in *Nicotiana benthamiana via* Agrobacterium-mediated transient gene expression, under the control of a strong constitutive plant promoter. We evaluated their ability to induce hypersensitive cell death (PCD) or suppress PCD triggered by the elicitin INF1. Potential apoplastic effectors with high cysteine content were cloned with signal peptide while potential intracellular effectors were also cloned without signal peptide to ensure proper localization after expression in plant cells. This way, one candidate effector (sr16441) was identified that is able to suppress cell death induced by the elicitin INF1.

## Materials and Methods

### Cloning of Candidate Effector Genes

**Figure 1:**
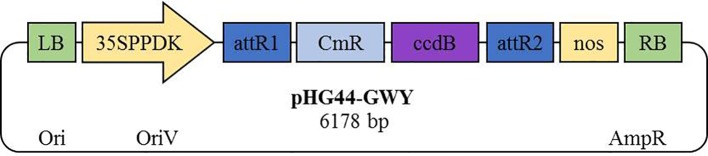
Principal elements of the Gateway-modified gene expression vector pHG44-GWY. The binary vector pHG44 was modified to carry a Gateway cloning cassette with the *ccdB* suicide gene and *attR* recombination sites along with the promoter 35SPPDK and *nos* terminator between its left and right border repeats from nopaline C58 T-DNA.

Effector candidates were mined from a large collection of SRZ proteins predicted to be secreted and lacking functional annotation (Schirawski et al., 2010; Schweizer et al., 2018). SignalP 5.0 (Armenteros et al., 2019) was used to predict the location of putative secretion signal peptides. Amino acid sequences of mature (i.e. lacking their signal peptide) putative effector proteins were analyzed for their cysteine content using the webserver DIANNA (Ferrè and Clote, 2005). We selected 56 effector candidates for cloning, with, without, or both with and without predicted signal peptides, totaling 62 constructs (see **Table 1**). Gene-specific primer pairs (**Table S1**) were used in PCR amplification reactions with genomic DNA from SRZ isolates as template. Amplified genes were cloned into the binary plasmid pHG44-GWY. pHG44-GWY is a derivative of pHG44 that was modified by Gibson assembly (Gibson et al., 2009). The plasmid pHG44 in turn was generated from the plasmid pP35S:GFP-SAD1ΔSP-T35 (Ghareeb et al., 2015) by digesting it with *AscI* to produce a 6.5 kb fragment, which then was dephosphorylated using calf intestinal alkaline phosphatase (NEB). pP35S:mCherry-T35 plasmid was digested using *BssHII* and *MluI* to generate a 1.6 kb fragment containing the P35S:mCherry-T35 construct. Ligation of the two aforementioned fragments resulted in the pP35S:GFP-SAD1ΔSP-T35-P35S:mCherry-T35 expression vector (pHG44). A pair of primers containing 20 bp overhangs (**Table S1**) was used to amplify a segment of about 3 kb from pHG44 consisting of left and right border repeats from nopaline C58 T-DNA, the resistance cassette (Amp^R^) and origin of replication (ori and oriV). The amplified segment was then ligated with a 3 kb BsrBI fragment of the plasmid HBT-sGFP(S65T)-NOS supplied by Jen Sheen, Boston, USA (Cheng et al., 2001) containing the recombination sites *att*R1 and *att*R2 suited for Gateway cloning along with the 35S-PPDK hybrid promoter and *nos* terminator (**Figure 1**).

**Table 1:** SRZ genes selected to be tested for programmed cell death (PCD) suppression in *N. benthamiana* in this study. Fifty-six effector candidates were cloned from genomic DNA, some of them carry introns.

Gene	Amino acid identity SRZ-SRS	Cysteine content^A^	Likelihood for secretion^B^	Intron	Protein size (aa)^C^	Construct
sr02614	69,8	7	0.9907	Yes	212	pHG44-GWY_sr02614
sr10057	89,3	0	0.9557	No	206	pHG44-GWY_sr10057ΔSP
sr10069	85,0	0	0.9647	No	234	pHG44-GWY_sr10069
sr10077	85,0	0	0.9925	No	180	pHG44-GWY_sr10077
sr10314^D^	53,0	0	0.9036	No	228	pHG44-GWY_sr10314
sr10529	75,0	0	0.9029	Yes	117	pHG44-GWY_sr10529
sr10532	89,1	9	0.9896	No	636	pHG44-GWY_sr10532
sr10702	99,0	4	0.9849	No	595	pHG44-GWY_sr10702
sr10767	85,8	0	0.9963	No	120	pHG44-GWY_sr10767
						pHG44-GWY_sr10767ΔSP
sr11002.2	82,8	1	0.9934	Yes	187	pHG44-GWY_sr11002.2
sr11006	78,0	0	0.9591	Yes	173	pHG44-GWY_sr11006
sr11130	31,2	4	0.9979	Yes	174	pHG44-GWY_sr11130
sr11132	42,4	5	0.9960	Yes	177	pHG44-GWY_sr11132
sr11133	81,2	5	0.9926	Yes	191	pHG44-GWY_sr11133
sr11238	89,6	0	0.9927	No	395	pHG44-GWY_sr11238ΔSP
sr11352	83,9	0	0.9980	No	174	pHG44-GWY_sr11352ΔSP
sr11355	65,5	0	0.9798	Yes	206	pHG44-GWY_sr11355
sr11400	73,1	6	0.9990	No	175	pHG44-GWY_sr11400
sr11402^G^	66,4	3	0.2066	Yes	131	pHG44-GWY_sr11402
sr11947	65,3	0	0.9909	Yes	283	pHG44-GWY_sr11947
sr12084	84,3	8	0.9979	Yes	185	pHG44-GWY_sr12084
sr12085	53,4	8	0.9948	Yes	163	pHG44-GWY_sr12085
sr12538	78,5	7	0.9965	No	340	pHG44-GWY_sr12538
sr12897	91,9	4	0.9980	No	248	pHG44-GWY_sr12897
sr13367	63,7	0	0.9482	No	380	pHG44-GWY_sr13367x
						pHG44-GWY_sr13367ΔSP
sr13374	76,8	0	0.9982	No	309	pHG44-GWY_sr13374
sr13419	65,7	0	0.9961	No	190	pHG44-GWY_sr13419
sr13420	87,5	0	0.9965	No	183	pHG44-GWY_sr13420
						pHG44-GWY_sr13420ΔSP
sr13458	40,0	0	0.9517	No	175	pHG44-GWY_sr13458
						pHG44-GWY_sr13458ΔSP
sr13524	40,0	4	0.9844	Yes	139	pHG44-GWY_sr13524
sr13864^E^	8,7	1	0.9570	No	148	pHG44-GWY_sr13864
sr13897	99,0	0	0.9670	Yes	200	pHG44-GWY_sr13897
sr13901	69,8	0	0.9922	Yes	116	pHG44-GWY_sr13901
sr13903	94,9	1	0.9764	Yes	136	pHG44-GWY_sr13903
sr13904	88,1	0	0.9871	Yes	133	pHG44-GWY_sr13904
sr13905	98,4	0	0.8693	No	125	pHG44-GWY_sr13905
sr13906^F,G^	80,9	2	0.9551	No	141	pHG44-GWY_sr13906
sr14168	58,5	50	0.9206	Yes	1257	pHG44-GWY_sr14168
sr14220	86,5	0	0.9893	Yes	192	pHG44-GWY_sr14220
sr14221^D^	88,0	5	0.8339	No	217	pHG44-GWY_sr14221
sr14222	78,7	5	0.9629	No	258	pHG44-GWY_sr14222
sr14226	84,1	0	0.9975	Yes	232	pHG44-GWY_sr14226
sr14274	64,6	11	0.9957	No	757	pHG44-GWY_sr14274
sr14387	78,5	0	0.9083	No	274	pHG44-GWY_sr14387ΔSP
sr14685	88,7	0	0.9960	No	120	pHG44-GWY_sr14685
sr14941	88,9	0	0.9228	Yes	252	pHG44-GWY_sr14941
sr15147	77,7	4	0.9940	Yes	139	pHG44-GWY_sr15147
sr15149	87,1	4	0.9944	Yes	140	pHG44-GWY_sr15149
sr16247	81,5	3	0.9443	No	302	pHG44-GWY_sr16247
sr16441	64,5	0	0.9040	No	196	pHG44-GWY_sr16441
						pHG44-GWY_sr16441ΔSP
sr16553	86,9	0	0.8542	No	168	pHG44-GWY_sr16553
						pHG44-GWY_sr16553ΔSP
sr16558	87,3	0	0.9836	No	181	pHG44-GWY_sr16558
sr16561	38,4	0	0.8924	No	176	pHG44-GWY_sr16561
sr17138	2,2	0	0.9981	No	72	pHG44-GWY_sr17138
sr17609^H^	15,9	15	0.0016	Yes	437	pHG44-GWY_sr17609
sr20006	78,5	0	0.9988	Yes	171	pHG44-GWY_sr20006

^A^Cysteine content was analyzed after excluding the signal peptide.

^B^Likelyhood value for signal peptide prediction via the Sec/SP1 pathway as predicted by SignalP 5.0.

^C^Including the signal peptide.

^D^The predicted protein contains one transmembrane helix which should be removed with the signal peptide.

^E^The ORF was N-terminally extended by 80 amino acids based on homology with UMAG_00823 of U. maydis.

^F^The predicted protein retains one transmembrane helix after signal peptide removal which removes a second one.

^G^The gene was included because it occurred in a gene cluster encoding weakly conserved mainly secreted proteins.

^H^This gene lacking a secretion prediction was included as negative control.

All constructs were verified first by restriction digest using several enzymes, then by sequencing. A total of 25 out of 46 constructs cloned with signal peptide contain introns (**Table 1**). A total of 49 constructs (see **Figure S2**) were tested in TSL, Norwich, UK, for their capacity to induce or suppress cell death induced by INF1, under the supervision of Sophien Kamoun, using p35S_ ΔGFP (Chaparro-Garcia et al., 2015) as a negative control and pBIN-plus-Avr3a-KI (Chaparro-Garcia et al., 2015) as a positive control. The remaining constructs were evaluated at the RWTH Aachen University, where pHG44-GWY_GFP was used as negative control, and pBINplus-Avr3a-KI was used as a positive control. *A. tumefacien*s strains GV3101 containing the constructs pGR106-INF1 (Huitema et al., 2005) and pBINplus-Avr3a-KI (Chaparro-Garcia et al., 2015) were kindly provided by Sophien Kamoun. INF1 was amplified using the primer pairs indicated in **Table S1** and cloned in pHG44-GWY to test the efficiency of this plasmid.

### Growth of Microbial Strains and Cultivation of Plants

Recombinant binary plasmids were maintained and propagated in *Escherichia coli*, strain Top10, grown in lysogeny broth (LB) media supplemented with 50 µg/ml carbenicillin. Basic molecular cloning techniques were used (Maniatis et al., 1988).

Recombinant *A. tumefaciens* genotype GV3101pMP90RK carrying constructs in the pHG44-GWY vector were routinely grown in LB media with appropriate antibiotics (rifampicin 100 µg/ml, gentamycin 50 µg/ml, kanamycin 50 µg/ml, carbenicillin 50 µg/ml) at 28°C with shaking at 180 rpm overnight. Agrobacterium strains were transformed with plasmid vectors by heat shock using a protocol provided by DNA Cloning Service e. K. available online (http://www.dna-cloning.com/agrobacterium). Transformed cells were grown, collected by centrifugation (5000 rpm, 5 minutes, at room temperature), re-suspended in infiltration buffer (10 mM MgCl_2_, 10 mM MES, pH 5.6, and 200 mM acetosyringone), and incubated at room temperature for 1–2 hours before infiltration.

*N. benthamiana* plants were cultivated and maintained throughout the experiments in a plant growth chamber or greenhouse under 16/8 hour light/dark photoperiod at 22°C and high light intensity. The experiments were performed using leaves of 4 to 6 week-old plants.

### Cell Death and Cell Death Suppression Assays

All constructs were first tested whether they induce cell death. The known PCD elicitor psojNIP (Qutob et al., 2002) or pGR106-INF1 (Huitema et al., 2005) was used as a control. *A. tumefaciens* strains expressing the SRZ effectors or controls were grown to a final OD_600_ of 0.5 and used to infiltrate the abaxial leaf side of 4 to 6 week-old *N. benthamiana* plants using a 1-ml syringe. Induction of PCD was visually assessed at 3, 4, and 5 days after infiltration. For cell death suppression assays, the infiltration sites were challenged again after 24 hours with recombinant *A. tumefaciens* carrying pGR106-INF1 at a final OD_600_ of 0.2 as previously described (Huitema et al., 2004; Bos et al., 2006); GFP and Avr3a-KI served as negative and positive control, respectively. A suppressor of posttranscriptional gene silencing from *Tomato bushy stunt virus* (P19) known to increase gene expression in the agroinfiltration assay (Oh et al., 2009) was used to improve the expression of 13 constructs (see **Figures 3C, E**). Strains carrying the plasmid pBIN61-P19 (Voinnet et al., 1999) were mixed in induction buffer with strains carrying the candidate effectors in a ratio of 1:1 (final OD_600_ of 1) and coinfiltrated. Symptom development and possible suppression of PCD was monitored at 3, 4, and 5 days after the second infiltration (Bos et al., 2006; Oh et al., 2009). The degree of PCD of leaves (HR index) was scored on a previously described seven-point scale according to the size of the necrotic area (grade 0 when no necrosis is observed, grade 7 when necrosis is confluent) (Wu et al., 2017). Each treatment was assayed on two plants with three leaves for each plant. Therefore, at least five infiltration sites were evaluated for each treatment. The experiment done in Aachen was conducted at least three times, two times without pBIN61-P19. The number of infiltration sites showing PCD was counted for each construct. One-way ANOVA with post-hoc Tukey test or independent-samples t-tests was used for statistical analysis conducted in SPSS (IBM Corp. Released 2017. IBM SPSS Statistics for Windows, Version 25.0. Armonk, NY: IBM Corp.). The graphs (except those in the **Supplemental Material** available online) display results of the experiments when pBIN61-P19 was used.

## Results

We randomly selected a total of 82 putative effector proteins for PCR amplification of the respective open reading frames. After excluding genes with weak or unsuccessful PCR-amplification, we finally generated a library of 62 constructs using primers corresponding to 56 ORFs that were either cloned with (46 genes), without (four genes), or with and without (six genes) their predicted signal peptides (**Table 1**). Since apoplastic effectors often contain multiple cysteine residues (Stergiopoulos and de Wit, 2009), we made sure that the ten ORFs selected for cloning without signal peptide (and thus ending up within the plant cell after heterologous expression) encoded proteins with no or only one cysteine residue (**Table 1**). The amplicons were cloned under the control of the constitutive hybrid 35S-PPDK promoter suited for gene expression in *N. benthamiana* (**Figure 1**) and were sequenced prior to use. Of the 56 ORFs that were cloned, 53 had less than 95% amino acid conservation to the respective orthologs of *S. reilianum* f. sp. *reilianum*, 46 had less than 300 amino acids, and one did not have a secretion prediction and served as negative control (**Table 1**).

**Figure 2:**
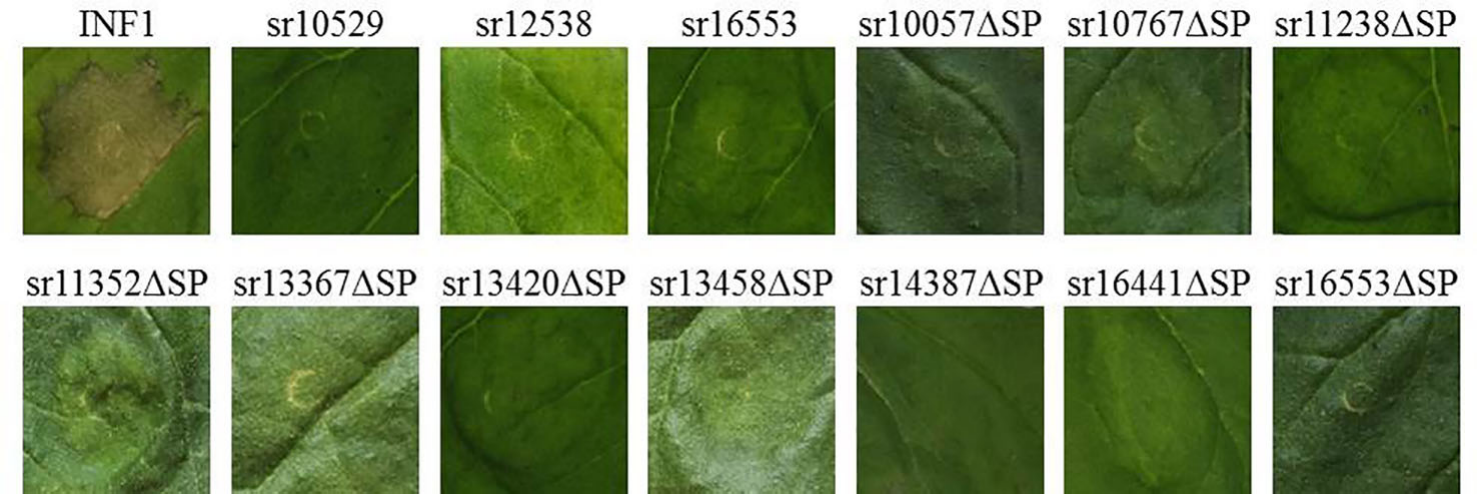
PCD induction assay. Agrobacterium strains carrying constructs with *Sporisorium reilianum* f. sp. *zeae* (SRZ) effectors were infiltrated in *N. benthamiana*. The results were evaluated at 3, 4, and 5 days after infiltration and compared to that of Infestin1 (INF1). None of the constructs could induce cell death under our experimental conditions. Pictures were taken at 4 days after infiltration.

To determine whether diverse *S. reilianum* f. sp. *zeae* effectors perturb host cellular processes, the 62 constructs were expressed in *N. benthamiana* using Agrobacterium-mediated transient transformation. This method has been shown to be a valuable initial screening tool to determine whether particular genes induce or suppress defense-associated PCD (Huitema et al., 2004; Oh et al., 2009). To verify that none of the candidate effectors induces cell death, *N. benthamiana* leaves were first infiltrated with *A. tumefaciens* strains carrying each of the 62 constructs or the controls psojNIP (Qutob et al., 2002) or pGR106-INF1 (Huitema et al., 2005) that encode known cell death-inducing elicitins. The infiltrated sites were visually evaluated at 3, 4, and 5 days after infiltration for signs of cell death. Phenotypic evaluation of the infiltrated sites revealed that only the known elicitins NIP of *P. sojae* and INF1 of *P. infestans* but none of the 62 constructs led to cell death induction (**Figure 2**; **Figure S1**, see **Supplemental Material** available online).

**Figure 3:**
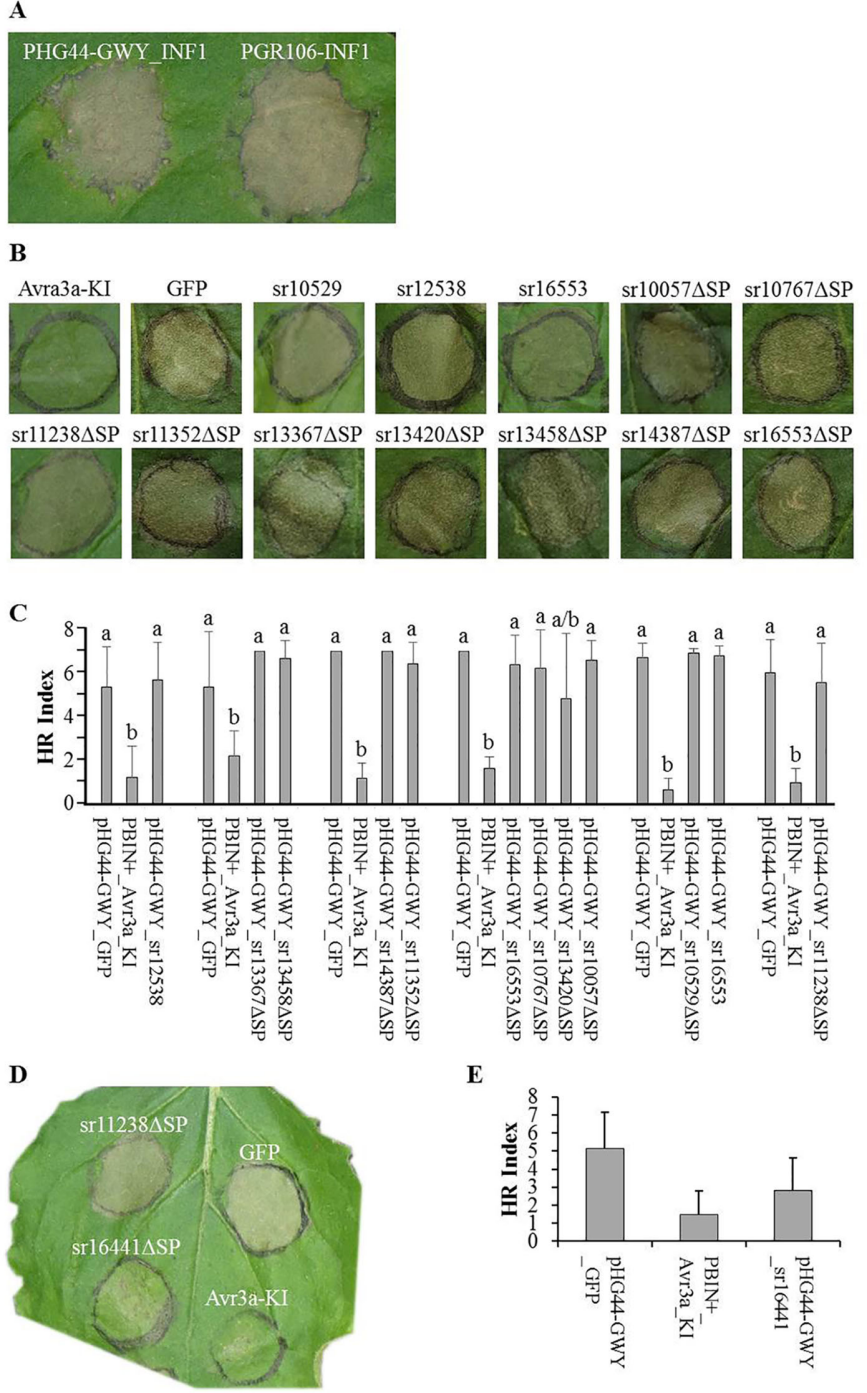
Cell death suppression assays. Agrobacterium strains carrying effectors were infiltrated in *N. benthamiana*. Candidate effectors from *Sporisorium reilianum* f. sp. *zeae* (SRZ) were infiltrated along with GFP (negative control) and Avr3a-KI (positive control), one day later the infiltration sites were challenged with the elicitin Infestin1 (INF1). The infiltration sites were evaluated after 3, 4, and 5 days. Pictures were taken at day 4. **(A)** Transient expression of both, pHG44-GWY-INF1 and pGR106-INF1 induced necrosis of the infiltrated area. Although necrosis induction by pHG44-GWY_INF1 was slightly weaker, we could show that expression of pHG44-GWY led to an expression strong enough to induce a cell-death response. **(B)** Leaves infiltrated with GFP and challenged with INF1 showed cell death, while leaves infiltrated with Avr3a-KI and challenged with INF1 did not show cell death. Furthermore, most of the constructs carrying *Sporisorium reilianum* f. sp. *zeae* (SRZ) effectors could not suppress INF1-induced cell death. **(C)** Quantitative comparison of the means of HR indexes of the infiltration sites of SRZ candidate effectors (co-infiltrated with pBIN61-P19) shows that they were statistically significantly higher then that from pBINplus-AVR3a-KI and had no significant difference to the means of pHG44-GWY-GFP, indicating that those candidate effectors are not able to suppress INF1-induced cell death. The experiment was conducted three times, two times without pBIN61-P19. Each column shows the mean and standard deviation. The letters above each column indicate statistically significant differences of the HR index (P < 0.01). **(D)** The strain carrying the predicted cytoplasmic effector sr16441ΔSP could suppress INF1-induced cell death. **(E)** Quantitative comparison shows that the mean of HR index of pHG44-GWY-sr16441ΔSP was statistically significantly lower than that of the pHG44-GWY-GFP control and was as low as that of pBINPLUS-AVR3a-KI (P < 0.01), indicating that sr16441ΔSP was able to suppress INF1-induced cell death. The experiments were repeated at least three times. The percentage was calculated from 30 infiltration sites. Columns show the mean and standard deviation. Different letters above each column indicate statistically significant differences of the HR index (P < 0.01).

We wondered whether a lack of cell-death induction activity of the tested constructs was a result of non-sufficient mRNA generation or wrong splicing of constructs that were cloned with introns. Therefore, we isolated total RNA of *N. benthamiana* leaves 4 days after infiltration with *A. tumefaciens* strains carrying one of seven constructs for expression of selected effectors, either with or without intron and either with or without signal peptide. Using RT-PCR, we could show that all seven constructs were expressed and that the three tested constructs with intron were correctly spliced (**Figure S2**, see **Supplemental Material** available online). Since all effector constructs were cloned in their native form without any tag that could be used in Western blot experiments, we decided to check protein expression by cloning the INF1 gene in the effector delivery vector pHG44-GWY. We compared the cell-death inducing activity of INF1 when expressed from pGR106-INF1 or from pHG44-GWY_INF1. Both constructs clearly induced necrosis of the infiltrated area (**Figure 3A**). Although necrosis induction by pHG44-GWY_INF1 was slightly weaker, this experiment showed that expression of pHG44-GWY led to protein expression strong enough to induce a cell-death response. Hence the pHG44-GWY vector was found to be suitable for the conducted experiment, suggesting that none of the tested constructs was able to induce cell death in *N. bentamiana*.

To test whether any of the cloned SRZ effectors had a function in suppressing INF1-induced cell death, we first infiltrated leaves of *N. benthamiana* with Agrobacteria delivering the effector constructs, and challenged the same area at 24 hours with Agrobacteria delivering the construct for expression of INF1. When first infiltrating the leaves with Agrobacteria delivering a construct for expression of AVR3a-KI, an effector of *P. infestans* that was shown to suppress INF1-induced cell death (Bos et al., 2006), a challenge with INF1-expressing Agrobacteria did not lead to cell death. In contrast, when first infiltrating the leaves with Agrobacteria delivering a construct for expression of GFP, challenging with INF1-expressing Agrobacteria led to clearly visible cell death (**Figure 3B**). Of the 62 tested constructs, 61 were not able to suppress INF1-induced cell death (**Figures 3B, C**; **Figure S3**, see **Supplemental Material** available online).

In contrast, delivery of the sr16441ΔSP expression construct efficiently prevented INF1-induced cell death (**Figures 3D, E**). We quantified the amount of induced necrosis within the infiltrated leaf area (Wu et al., 2017). The necrosis ratio of pHG44-GWY-sr16441ΔSP was significantly lower than that of the pHG44-GWY-GFP control and was as low as that of pBINplus-AVR3a-KI (P < 0.01) (**Figure 3E**). We verified the cell-death suppression activity of sr16441ΔSP by infiltration of pHG44-GWY-sr16441ΔSP together with p19 (Oh et al., 2009), which led to even clearer cell death suppression response (not shown). Interestingly, using pHG44-GWY-sr16441, which leads to expression of sr16441 including its putative signal peptide, did not lead to suppression of INF1-induced cell death (**Figure S3**, see **Supplemental Material** available online). This result suggests that cytoplasmically expressed sr16441ΔSP can consistently suppress PCD induced by INF1 in *N. benthamiana*.

## Discussion

In this study, we evaluated the ability of 62 expression constructs for putative effectors of SRZ to induce cell death or suppress INF1-induced cell death in *N. benthamiana*. None of the tested constructs induced cell death, but sr16441 suppressed the cell death induced by INF1 when expressed without signal peptide.

Suppression of plant innate immunity is an important function of plant pathogens during plant cell invasion (Hogenhout et al., 2009). AVR3a-KI, a *P. infestans* host-translocated (cytoplasmic) effector, deregulates plant immune signaling leading to suppression of the cell death triggered by several pathogen molecules, among them the PAMP-like elicitin INF1 of *P. infestans* (Bos et al., 2006; Bos et al., 2010; Gilroy et al., 2011). To suppress PCD triggered by INF1, Avr3a-KI interacts with and stabilizes the host ubiquitin E3 ligase CMPG1, which is required for INF1-dependent cell death. Stabilization of CMPG1 by AVR3a consists of modifying CMPG1 activity, preventing the normal 26S proteasome-dependent degradation of itself and potentially of its protein substrates in the host cell. Thus, AVR3a blocks signal transduction cascades initiated at the plasma membrane after pathogen perception (Bos et al., 2010). Currently, over 30 effectors from four different oomycete species are known to suppress INF1-triggered responses, however, knowledge on how elicitin-triggered responses are suppressed is currently limited to AVR3a-KI (Derevnina et al., 2016).

Cell death-inducing effectors have been identified in the *Ustilago hordei*–barley pathosystem that is genetically ruled by a “gene-for-gene” interaction. One of the avirulence proteins, UhAvr1, induces local cell death during an incompatible interaction with barley, in the presence of Ruh1 (Ali et al., 2014). Incompatible interaction between SRZ and sorghum leads to induction of phytoalexins, which culminates in cell death at the site of infection (Zuther et al., 2012). Hence, the existence of avirulence effectors in SRZ that would induce cell death in *N. benthamiana* would be plausible. It is possible that cell death-inducing effectors are among the majority of effectors that were not tested, or that the used constructs did not lead to protein expression. Also, the used method would only identify cell death effectors targeting conserved host proteins, while most effectors may induce cell death only in one particular plant species. Therefore, testing the selected effectors for cell death induction in maize or sorghum might prove rewarding.

The putative effector sr16441 that we identified as suppressing INF1-induced cell death in *N. benthamiana*, is a predicted small protein of 196 amino acids excluding its signal peptide. The protein shows only 65% identity to its closest homolog SRS1_16441 of *Sporisorium reilianum* f. sp. *reilianum*. The protein has weakly conserved homologs also in *Sporisorium scitamineum* (SPSC_01549 and SPSC_1550) and *Sporisorium graminicola* (EX895_003212). In how far the proteins fulfill the same or a similar function in their respective host plants needs to be elucidated. The candidate effector sr16441 does not have any recognizable domains but has a clear prediction for secretion. In *N. benthamiana*, it was able to suppress INF1-induced cell death only when expressed inside the plant cell, i.e. without its signal peptide. This indicates that its signal peptide is functional and that for INF1-induced cell death suppression, an intracellular localization is necessary. Supposing that sr16441 also functions in cell death suppression in maize when secreted by *S. reilianum*, the protein would need to be taken up by the plant cell and target a factor that is conserved between *N. benthamiana* and Maize.

Further investigations are needed to verify whether sr16441 contributes to virulence, host selection, or symptom formation of *S. reilianum* on maize and sorghum. Gene expression analysis, localization of the protein after secretion by the fungus, and a possible function in suppressing cell death in sorghum or maize should be tested. However, a possible screening system would depend on the identification of a factor that reliably induces cell death in sorghum or maize. Although there is still a lot to do to elucidate the function of sr16441 in its natural system, the conducted assay revealed a possible function for this candidate effector that can now be elucidated in detail.

## Data Availability Statement

The datasets generated for this study are available on request to the corresponding author.

## Author Contributions

JS and DD conceived the study. DD did the cloning, cell-death and cell death-suppression experiments. NA did the effector expression control. DD and JS wrote the manuscript and prepared the figures. HG generated pHG44. All authors read and approved the submitted manuscript version.

## Funding

DD was funded by the mobility program for Brazilian students “Science without Borders” (Ciência sem Fronteiras). NA was supported by the German Academic Exchange Service (DAAD). JS received funding from the German Research Foundation (DFG), as well as institutional support by the RWTH Aachen University.

## Conflict of Interest

The authors declare that the research was conducted in the absence of any commercial or financial relationships that could be construed as a potential conflict of interest.
