# Supplementary material for: Screening of Secreted Proteins of Sporisorium reilianum f. sp. zeae for Cell Death Suppression in Nicotiana benthamiana
Source: Front Plant Sci. 2020 Feb 19;11:95. doi: 10.3389/fpls.2020.00095 (PMC7042202; doi:10.3389/fpls.2020.00095)
Supplement: Supplementary file 1 [file DataSheet_1.pdf]

Supplemental Material

Screening of secreted proteins of *Sporisorium reilianum* f. sp. *zeae* for cell death suppression in *Nicotiana benthamiana*

Deiziane Dutra[1], Nisha Agrawal[1,4], Hassan Ghareeb[2,3,*], and Jan Schirawski[1,3,4,§]

[1] Microbial Genetics, Institute of Applied Microbiology, RWTH Aachen University, Aachen, Germany

[2] Plant Biotechnology, National Research Centre, 12311 Cairo, Egypt

[3] Molecular Biology of Plant-Microbe Interactions, Albrecht-von-Haller Institute of Plant Sciences, Schwann-Schleiden Research Center, Georg-August-University Göttingen, Julia-Lermontowa-Weg 3, 37077 Göttingen, Germany

[4] Genetics, Matthias-Schleiden-Institute, Friedrich-Schiller-University Jena, Philosophenweg 12, 07743 Jena, Germany

* Present Address: Plant Cell Biology, Albrecht-von-Haller Institute of Plant Sciences, Georg-August-University Göttingen, Julia-Lermontowa-Weg 3, 37077 Göttingen, Germany

§ Author for correspondence:

Jan Schirawski

jan.schirawski@uni-jena.de

Contents:

Supplemental Tables

Table S1: Oligonucleotides used in this study. ……………………………….. Page 2

Supplemental Figures

Figure S1: PCD induction assay. ……………………………………………… Page 11

Figure S2: Expression and demonstration of splicing of selected candidate effectors in *N. benthamiana* as tested by RT-PCR. …………………………… Page 12

Figure S3: PCD suppression assay. …………………………………………… Page 13

Table S1. Oligonucleotides used in this study. Oligonucleotides used for cloning contain the attachment sites (*attB1* for forward primer and *attB2* for reverse primer) necessary for Gateway cloning. Oligonucleotides used for Gibson assembly contain a 20 bp overhang.

Gene of interest	Plasmid construct	Primer name	Primers for cloning
sr10069	pHG44-GWY_sr10069	oKC3	GGGGACAAGTTTGTACAAAAAAGCAGGCTTCGAAGGAGATAGAACCATGGTGCACTCACCTCG
		oKC4	GGGGACCACTTTGTACAAGAAAGCTGGGTGCTATGGCTCAACGATGCGTAG
sr10077	pHG44-GWY_sr10077	oKC5	GGGGACAAGTTTGTACAAAAAAGCAGGCTTCGAAGGAGATAGAACCATGAATCTTCTGCCCTTCAAAC
		oKC6	GGGGACCACTTTGTACAAGAAAGCTGGGTGCTATACTGATAAATGGAGAGCAGG
sr10529	pHG44-GWY_sr10529	oKC9	GGGGACAAGTTTGTACAAAAAAGCAGGCTTCGAAGGAGATAGAACCATGCTGGTCCACTCG
		oKC10	GGGGACCACTTTGTACAAGAAAGCTGGGTGTCAGTGGCTCTTGTACCC
sr13458	pHG44-GWY_sr13458	oKC11	GGGGACAAGTTTGTACAAAAAAGCAGGCTTCGAAGGAGATAGAACCATGAAGCTCACCGTATTCAAGTTCG
		oKC12	GGGGACCACTTTGTACAAGAAAGCTGGGTGCTAGGCTGCAGAGGCAGGCATC
sr13524	pHG44-GWY_sr13524	oKC13	GGGGACAAGTTTGTACAAAAAAGCAGGCTTCGAAGGAGATAGAACCATGAAGATCCAAACCGCCCTG
		oKC14	GGGGACCACTTTGTACAAGAAAGCTGGGTGCTACTTCCACACATAGTCCG
sr16441	pHG44-GWY_sr16441	oKC21	GGGGACAAGTTTGTACAAAAAAGCAGGCTTCGAAGGAGATAGAACCATGCGGTTCTCGGTCAGCTC
		oKC22	GGGGACCACTTTGTACAAGAAAGCTGGGTGCTAGCGGCCCCAAAGGTTTATC
sr11947	pHG44-GWY_sr11947	oKC23	GGGGACAAGTTTGTACAAAAAAGCAGGCTTCGAAGGAGATAGAACCATGAAGCTCCTCGCATCCTTCG
		oKC24	GGGGACCACTTTGTACAAGAAAGCTGGGTGTCACACCAGACCTTGTTGGCTAGAG
sr13419	pHG44-GWY_sr13419	oKC27	GGGGACAAGTTTGTACAAAAAAGCAGGCTTCGAAGGAGATAGAACCATGCGGTCCACTCTCCACCTCTTC

		oKC28	GGGGACCACTTTGTACAAGAAAGCTGGGTGCTACCATGCGAGATTGCTCGTGATCC
sr11402	pHG44-GWY_sr11402	oKC29	GGGGACAAGTTTGTACAAAAAAGCAGGCTTCGAAGGAGATAGAACCATGAAGATGCACAGCGGC
		oKC30	GGGGACCACTTTGTACAAGAAAGCTGGGTGTCAAGTCTTCCACAGCACGTC
sr14168	pHG44-GWY_sr14168	oKC15_new	GGGGACAAGTTTGTACAAAAAAGCAGGCTTCGAAGGAGATAGAACCCTTGCCAACCTCGAAGCTAC
		oKC16_new	GGGGACCACTTTGTACAAGAAAGCTGGGTGTCGAGCGATCAAAGAGACAG
sr11400	pHG44-GWY_sr11400	oKC31	GGGGACAAGTTTGTACAAAAAAGCAGGCTTCGAAGGAGATAGAACCATGAAGATCAACAACGCCCTCC
		oKC32	GGGGACCACTTTGTACAAGAAAGCTGGGTGTCAGCACTTGACCAGGTCATGCTC
sr15147	pHG44-GWY_sr15147	oKC33	GGGGACAAGTTTGTACAAAAAAGCAGGCTTCGAAGGAGATAGAACCATGCGAAACTCATTCTTTCTCGTGTC
		oKC34	GGGGACCACTTTGTACAAGAAAGCTGGGTGCTAGATGATCTGAGGGGCAGGG
sr16247	pHG44-GWY_sr16247	oKC41	GGGGACAAGTTTGTACAAAAAAGCAGGCTTCGAAGGAGATAGAACCATGCTTACCAAGTACGCCGTCCTC
		oKC42	GGGGACCACTTTGTACAAGAAAGCTGGGTGTCAGGCTGAGTAGATGCGGTCTGC
sr12084	pHG44-GWY_sr12084	oKC45	GGGGACAAGTTTGTACAAAAAAGCAGGCTTCGAAGGAGATAGAACCATGGTTCAGCTCAAGTCGTCGTTC
		oKC46	GGGGACCACTTTGTACAAGAAAGCTGGGTGCTAGGGCTCGTCGGCCTTCTTGG
sr10767	pHG44-GWY_sr10767	oKC51	GGGGACAAGTTTGTACAAAAAAGCAGGCTTCGAAGGAGATAGAACCATGAAGTCTTCATTCTTGCTCCC
		oKC52	GGGGACCACTTTGTACAAGAAAGCTGGGTGTCACTGTCTTGCCGGACG
sr16553	pHG44-GWY_sr16553	oKC53	GGGGACAAGTTTGTACAAAAAAGCAGGCTTCGAAGGAGATAGAACCATGAAGATTCGAACTTTGACGGTCTTG
		oKC54	GGGGACCACTTTGTACAAGAAAGCTGGGTGCTAGGCAACATTTCTGGCTTGCTTAG

sr17149	pHG44-GWY_sr17149	oKC55	GGGGACAAGTTTGTACAAAAAAGCAGGCTTCGAAGGAGATAGAACCATGCGAAGCCTCAACTTTATC
		oKC56	GGGGACCACTTTGTACAAGAAAGCTGGGTGTTAAATCCAGATCGCTCTC
sr16558	pHG44-GWY_sr16558	oKC59	GGGGACAAGTTTGTACAAAAAAGCAGGCTTCGAAGGAGATAGAACCATGAAACAATTTCGTCTGATGGCCCTGGCG
		oKC60	GGGGACCACTTTGTACAAGAAAGCTGGGTGCTAGCCACGCATCCTTTGAATGACG
sr13420	pHG44-GWY_sr13420	oKC61	GGGGACAAGTTTGTACAAAAAAGCAGGCTTCGAAGGAGATAGAACCATGGCCGACATACGTCGACAC
		oKC62	GGGGACCACTTTGTACAAGAAAGCTGGGTGTCATTGTAAAGGAAGAGGATGG
sr14941	pHG44-GWY_sr14941	oKC63	GGGGACAAGTTTGTACAAAAAAGCAGGCTTCGAAGGAGATAGAACCATGTTGGTGCGTTTCACAACGCTTGC
		oKC64	GGGGACCACTTTGTACAAGAAAGCTGGGTGTTAGGGCTTGAATTTCCTCACGTACTC
sr13897	pHG44-GWY_sr13897	oKC64	GGGGACCACTTTGTACAAGAAAGCTGGGTGTTAGGGCTTGAATTTCCTCACGTACTC
		oKC72	GGGGACCACTTTGTACAAGAAAGCTGGGTGTTAGGCAGAGTCTGCAATGG
sr13903	pHG44-GWY_sr13903	oKC77	GGGGACAAGTTTGTACAAAAAAGCAGGCTTCGAAGGAGATAGAACCCCGTCTTCACCAACAATGTCTC
		oKC78	GGGGACCACTTTGTACAAGAAAGCTGGGTGGCGCTAAGTGCTGCAGTCTC
sr13905	pHG44-GWY_sr13905	oKC79	GGGGACAAGTTTGTACAAAAAAGCAGGCTTCGAAGGAGATAGAACCAGCGCAACTCTGCAACAACAGC
		oKC80	GGGGACCACTTTGTACAAGAAAGCTGGGTGAAGCAGAGCCTAAGCAGAGAAG
sr14274	pHG44-GWY_sr14274	oKC89	GGGGACAAGTTTGTACAAAAAAGCAGGCTTCGAAGGAGATAGAACCCTGGCAAGTCTTTCAAGTCGACAC
		oKC90	GGGGACCACTTTGTACAAGAAAGCTGGGTGACGAGCAAGCCGTTCTGAGTACC

sr11006	pHG44-GWY_sr11006	oKC107	GGGGACAAGTTTGTACAAAAAAGCAGGCTTCGAAGGAGATAGAACCAGGCCTAGACACCTTTAGCACAAC
		oKC108	GGGGACCACTTTGTACAAGAAAGCTGGGTGGATCACCAACGATCTGGCTTACTC
sr11002.2	pHG44-GWY_sr11002.2	oKC99	GGGGACAAGTTTGTACAAAAAAGCAGGCTTCGAAGGAGATAGAACCATGCGTTCAACCTTCCACATCC
		oKC100	GGGGACCACTTTGTACAAGAAAGCTGGGTGCTTACTACCACCCGTAGTGGTTATG
sr14226	pHG44-GWY_sr14226	oKC101	GGGGACAAGTTTGTACAAAAAAGCAGGCTTCGAAGGAGATAGAACCAATGTCGCCTTTCCACCTTCTC
		oKC102	GGGGACCACTTTGTACAAGAAAGCTGGGTGCACGCCTTACACCTAAACATGC
sr14685	pHG44-GWY_sr14685	oKC103	GGGGACAAGTTTGTACAAAAAAGCAGGCTTCGAAGGAGATAGAACCCCAATCATGCTCTCGCATCTACC
		oKC104	GGGGACCACTTTGTACAAGAAAGCTGGGTGGAGCGTCTAGGCCAGATACTCG
sr02614	pHG44-GWY_sr02614	oNR131	GGGGACAAGTTTGTACAAAAAAGCAGGCTTCGAAGGAGATAGAACCATGTCGACCACTCAGCG
		oNR132	GGGGACCACTTTGTACAAGAAAGCTGGGTGTTAGTTGGCCTTGGGCTTCTCTTTCC
sr11130	pHG44-GWY_sr11130	oNR75	GGGGACAAGTTTGTACAAAAAAGCAGGCTTCGAAGGAGATAGAACCATGCTGATGTCAAACCGATCG
		oNR76	GGGGACCACTTTGTACAAGAAAGCTGGGTGTCAGAAATCGGACAGGTCGAC
sr11133	pHG44-GWY_sr11133	oNR121	GGGGACAAGTTTGTACAAAAAAGCAGGCTTCGAAGGAGATAGAACCATGTCGACCCAGAAGCC
		oNR122	GGGGACCACTTTGTACAAGAAAGCTGGGTGCTACTTCTTCTTCTTGACC
sr13367	pHG44-GWY_sr13367	oKC19	GGGGACAAGTTTGTACAAAAAAGCAGGCTTCGAAGGAGATAGAACCATGTTGGTAACAGCTATCATGC
		oKC20	GGGGACCACTTTGTACAAGAAAGCTGGGTGCTATTTTGGCCCTTGGGAAGC
sr13374	pHG44-GWY_sr13374	oNR133	GGGGACAAGTTTGTACAAAAAAGCAGGCTTCGAAGGAGATAGAACCATGTCTGTCTCGACCACGAAGC

		oNR134	GGGGACCACTTTGTACAAGAAAGCTGGGTGTCAAGCGCGGGCATGGG
sr13864	pHG44-GWY_sr13864	oNR113	GGGGACAAGTTTGTACAAAAAAGCAGGCTTCGAAGGAGATAGAACCATGGCTGTCAAAGCCGACC
		oNR114	GGGGACCACTTTGTACAAGAAAGCTGGGTGTCAAAACTGGAAAGCCAGC
sr13906	pHG44-GWY_sr13906	oNR155	GGGGACAAGTTTGTACAAAAAAGCAGGCTTCGAAGGAGATAGAACCATGCACCCACGTCGAGCATCAGC
		oNR156	GGGGACCACTTTGTACAAGAAAGCTGGGTGTCACGTTGCTCCCGACTTTG
sr14220	pHG44-GWY_sr14220	oNR163	GGGGACAAGTTTGTACAAAAAAGCAGGCTTCGAAGGAGATAGAACCATGCTCTTCAAGGCTCAAGC
		oNR164	GGGGACCACTTTGTACAAGAAAGCTGGGTGTCAATCGGTATCACCATCACTTTCC
sr16561	pHG44-GWY_sr16561	oNR91	GGGGACAAGTTTGTACAAAAAAGCAGGCTTCGAAGGAGATAGAACCATGCAGATTCAGCGACTCGTCACC
		oNR92	GGGGACCACTTTGTACAAGAAAGCTGGGTGTCATACCGCTTCGCGCACAACC
sr17138	pHG44-GWY_sr17138	oNR93	GGGGACAAGTTTGTACAAAAAAGCAGGCTTCGAAGGAGATAGAACCATGAAGTGCTACCTCGTCGTTGTCG
		oNR94	GGGGACCACTTTGTACAAGAAAGCTGGGTGTCAGTGAAGAGCCGAAGTGTCG
sr20006	pHG44-GWY_sr20006	oNR135	GGGGACAAGTTTGTACAAAAAAGCAGGCTTCGAAGGAGATAGAACCATGAAGTTCTTCCAAATCCTCATTGC
		oNR136	GGGGACCACTTTGTACAAGAAAGCTGGGTGTTAGAACCAGCCTGAGCTCG
sr14222	pHG44-GWY_sr14222	oNR151	GGGGACAAGTTTGTACAAAAAAGCAGGCTTCGAAGGAGATAGAACCATGTTCAGCCGCAACAAGTCC
		oNR152	GGGGACCACTTTGTACAAGAAAGCTGGGTGTCAGATACCACGGCCGTCGTC
sr11355	pHG44-GWY_sr11355	oNR127	GGGGACAAGTTTGTACAAAAAAGCAGGCTTCGAAGGAGATAGAACCATGAAGCTCAACGCCTAC
		oNR128	GGGGACCACTTTGTACAAGAAAGCTGGGTGCTACCAGAACCAGCTGACC

sr11132	pHG44-GWY_sr11132	oNR77	GGGGACAAGTTTGTACAAAAAAGCAGGCTTCGAAGGAGATAGAACCATGATGGCCACTCAATTAGCTGTG
		oNR78	GGGGACCACTTTGTACAAGAAAGCTGGGTGCTACTCCTTGTCCAAACGAATCC
sr12085	pHG44-GWY_sr12085	oNR123	GGGGACAAGTTTGTACAAAAAAGCAGGGGACAAGTTTGTACAAAAAAGCACATGTTCAAGCCCTC
		oNR124	GGGGACCACTTTGTACAAGAAAGCTGGGTCTCAAGGGGTATGTGATCTCT
sr13901	pHG44-GWY_sr13901	oNR119	GGGGACAAGTTTGTACAAAAAAGCAGGCTTCGAAGGAGATAGAACCATGAAGCTACTCATGGTCC
		oNR120	GGGGACCACTTTGTACAAGAAAGCTGGGTGTCAGGGATGGGCCTTTGAC
sr13904	pHG44-GWY_sr13904	oNR159	GGGGACAAGTTTGTACAAAAAAGCAGGCTTCGAAGGAGATAGAACCATGACATCTTTAGGATTTGCTCGGG
		oNR160	GGGGACCACTTTGTACAAGAAAGCTGGGTGCTATCGGCCTTGTTGACCC
sr14221	pHG44-GWY_sr14221	oKC83	GGGGACAAGTTTGTACAAAAAAGCAGGCTTCGAAGGAGATAGAACCGCAGCGATGATCGCTCAAAC
		oKC84	GGGGACCACTTTGTACAAGAAAGCTGGGTGTCAAGGCCTCTTACTAGGCTGTC
sr10702	pHG44-GWY_sr10702	oNR177	GGGGACAAGTTTGTACAAAAAAGCAGGCTTCGAAGGAGATAGAACCATGAAGTCGAGCTTCGTCG
		oNR178	GGGGACCACTTTGTACAAGAAAGCTGGGTGTCACTTCTGGCACTTGTTCC
sr12897	pHG44-GWY_sr12897	oDD_52	GGGGACAAGTTTGTACAAAAAAGCAGGCTTCGAAGGAGATAGAACCATGAAGTTCGCCTTCGGC
		oDD_53	GGGGACCACTTTGTACAAGAAAGCTGGGTGCTAACCCGAGGTGTTGATGTC
sr10314	pHG44-GWY_sr10314	oKC7	GGGGACAAGTTTGTACAAAAAAGCAGGCTTCGAAGGAGATAGAACCATGGCGCTTCGACGGAACTTG
		oKC8	GGGGACCACTTTGTACAAGAAAGCTGGGTGTCAAACGTTCGAAGGTGCATGAC
sr10532	pHG44-GWY_sr10532	oKC65	GGGGACAAGTTTGTACAAAAAAGCAGGCTTCGAAGGAGATAGAACCATGCGAACCTCTCCCGCTCTG

		oKC66	GGGGACCACTTTGTACAAGAAAGCTGGGTGTTAAGCCGAGTCCACGTCAG
sr17609	pHG44-GWY_sr17609	oKC81	GGGGACAAGTTTGTACAAAAAAGCAGGCTTCGAAGGAGATAGAACCCACCCAAACATCCATTTACG
		oKC82	GGGGACCACTTTGTACAAGAAAGCTGGGTGGTCGCTTTGCGTGGAGATTC
sr12538	pHG44-GWY_sr12538	oKC95	GGGGACAAGTTTGTACAAAAAAGCAGGCTTCGAAGGAGATAGAACCGACCCAATCCAGCAAGTCAGAG
		oKC96	GGGGACCACTTTGTACAAGAAAGCTGGGTGAGCGAGCTTGGCGACATCAG
sr11238Δsp	pHG44-GWY_sr11238Δsp	oBF9	GGGGACAAGTTTGTACAAAAAAGCAGGCTTCGAAGGAGATAGAACCATGGCCCCCACCGGTGATCAAG
		oNR168	GGGGACCACTTTGTACAAGAAAGCTGGGTGTCAAGGCTTACGGATCTTGC
sr16441Δsp	pHG44-GWY_sr16441Δsp	oBF26	GGGGACAAGTTTGTACAAAAAAGCAGGCTTCGAAGGAGATAGAACCATGGCGCCCATGTTCAAGTACC
		oKC22	GGGGACCACTTTGTACAAGAAAGCTGGGTGCTAGCGGCCCCAAAGGTTTATC
sr13367Δsp	pHG44-GWY_sr13367Δsp	oBF13	GGGGACAAGTTTGTACAAAAAAGCAGGCTTCGAAGGAGATAGAACCATGGCTCCCATGTGGAACAAC
		oKC20	GGGGACCACTTTGTACAAGAAAGCTGGGTGCTATTTTGGCCCTTGGGAAGCTC
sr13458Δsp	pHG44-GWY_sr13458Δsp	oBF18	GGGGACAAGTTTGTACAAAAAAGCAGGCTTCGAAGGAGATAGAACCATGGTGCTCAAGTTTGGTGCTGGCATC
		oKC12	GGGGACCACTTTGTACAAGAAAGCTGGGTGCTAGGCTGCAGAGGCAGGCATC
sr14387Δsp	pHG44-GWY_sr14387Δsp	oBF24	GGGGACAAGTTTGTACAAAAAAGCAGGCTTCGAAGGAGATAGAACCATGCCTTTGGTACCTGGAAGCTTCG
		oKC36	GGGGACCACTTTGTACAAGAAAGCTGGGTGCTACTGACCACGGCCCCAAAG
sr11352Δsp	pHG44-GWY_sr11352Δsp	oBF11	GGGGACAAGTTTGTACAAAAAAGCAGGCTTCGAAGGAGATAGAACCATGGCCCCAGTGCCTATGGCATC

		oKC44	GGGGACCACTTTGTACAAGAAAGCTGGGTGCTAGTCCTTCTGCTTTCCACTGCCG
sr16553Δsp	pHG44-GWY_sr16553Δsp	oBF28	GGGGACAAGTTTGTACAAAAAAGCAGGCTTCGAAGGAGATAGAACCATGGTTCAATTCACGAAAGAGTACAGTC
		oKC54	GGGGACCACTTTGTACAAGAAAGCTGGGTGCTAGGCAACATTTCTGGCTTGCTTAG
sr10767Δsp	pHG44-GWY_sr10767Δsp	oBF8	GGGGACAAGTTTGTACAAAAAAGCAGGCTTCGAAGGAGATAGAACCATGGCCCCAAGAGGAGCCCTCTTC
		oKC52	GGGGACCACTTTGTACAAGAAAGCTGGGTGTCACTGTCTTGCCGGACG
sr13420Δsp	pHG44-GWY_sr13420Δsp	oBF17	GGGGACAAGTTTGTACAAAAAAGCAGGCTTCGAAGGAGATAGAACCATGGAGACACCTCTAGAGGGAAGAG
		oKC62	GGGGACCACTTTGTACAAGAAAGCTGGGTGTCATTGTAAAGGAAGAGGATGG
sr10057Δsp	pHG44-GWY_sr10057Δsp	oBF1	GGGGACAAGTTTGTACAAAAAAGCAGGCTTCGAAGGAGATAGAACCATGTGGGGAAAACATCCAAAGTATC
		oKC2	GGGGACCACTTTGTACAAGAAAGCTGGGTGTTACAGAGGAAACCGGGGGAAG
GFP	pHG44_GWY_GFP	oNR103	GGGGACAAGTTTGTACAAAAAAGCAGGCTTCGAAGGAGATAGAACCATGGTGAGCAAGGGCGAGG
		oNR104	GGGGACCACTTTGTACAAGAAAGCTGGGTGTTACTTGTACAGCTCGTCC
INF1	pHG44_GWY_INF1	oDD_70	GGGGACAAGTTTGTACAAAAAAGCAGGCTCAATGGGATTTGTTCTCTTTTCAC
		oDD_24	GGGGACCACTTTGTACAAGAAAGCTGGGTATCATAGCGACGCACACGTAGAC
**Amplification**	**Description**	**Primer name**	**Primers for Gibson assembly**
pHG44 backbone	Forward	oDD_05	CTGTGTGAAATTGTTATCCGTGACAGGATATATTGGCGGGTAAAC
pHG44 backbone	Reverse	oDD_06	TACATTCAAATATGTATCCGCGTCCGCAATGTGTTATTAAGTTG
**cDNA Amplification**	**Description**	**Primer name**	**Primers for expression analysis**
sr14220	Forward	oNA226	GAAGTATGCCAACGCTACTG
	Reverse	oNA227	TCGGAGTAGGACATAGAGAC
sr14226	Forward	oNA238	CATCCTGCGTACATCAGCC

	Reverse	oNA239	CGCGTTCTTCATCTCCACC
sr10529	Forward	oNA270	GCCTCGTGCTCCATGTTCA
	Reverse	oNA233	GCCTCCGAAGTCGAAGAAC
sr16441Δsp and sr16441	Forward	oNA230	CGTCCAAGATTCGCACATTC
	Reverse	oNA231	GATCTGGTCAGAGGTCAATG
sr10767Δsp and sr10767	Forward	oNA228	GAGGAAGCTGCCAAGGATG
	Reverse	oNA229	TCGTTCGAGTCGTCTGAAG

Supplemental Figures

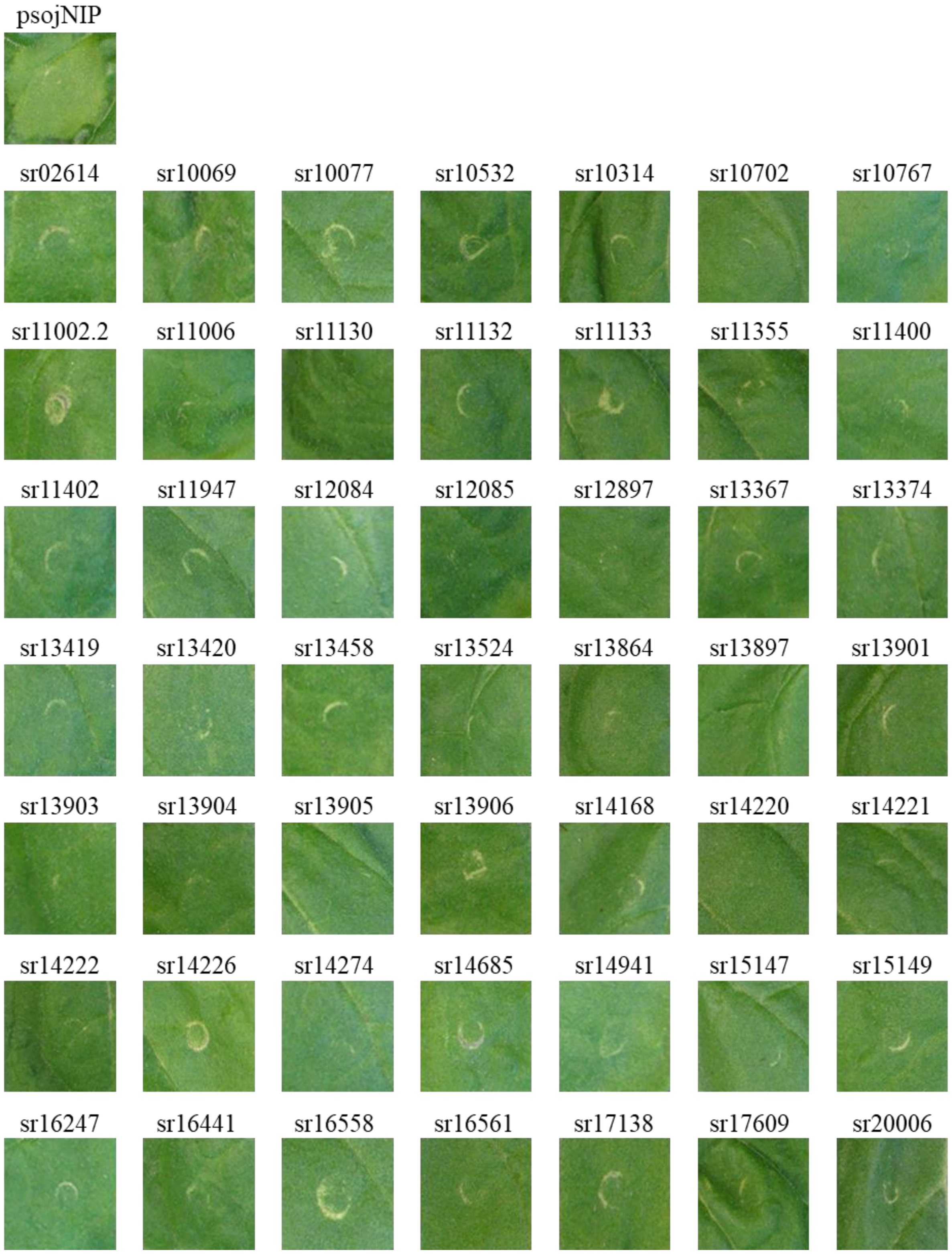

Figure S1. **PCD induction assay.** The assay was conducted at TSL, UK. *Agrobacterium* strains carrying constructs with SRZ effectors were infiltrated in *N. benthamiana* along with the control psojNIP and evaluated 3-5 days later. None of the constructs could induce cell death under the tested conditions. The picture was taken at 4 days after infiltration.

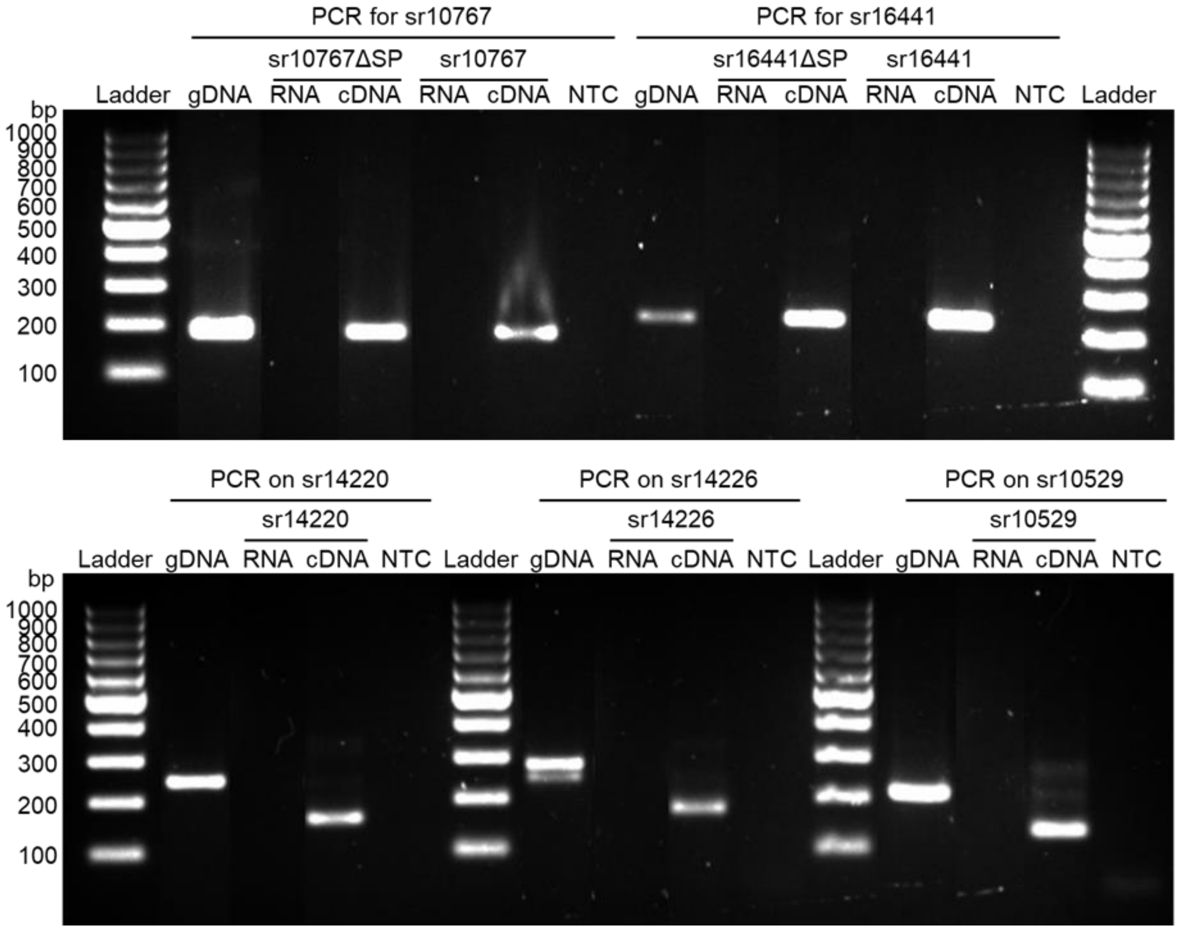

Figure S2. Expression and demonstration of splicing of selected candidate effectors in *N. benthamiana* as tested by RT-PCR. *N. benthamiana* leaves were infiltrated with *A. tumefaciens* transferring gene expression constructs for the indicated candidate effectors. At 4 dpi, total RNA was extracted, treated with DNase, and used for cDNA generation. PCR was done on RNA after DNase treatment (RNA), on cDNA (cDNA) and on genomic DNA of *S. reilianum* (gDNA) as positive control. The no template control (NTC) contained water instead of template. The samples were run on a 1.7% TAE agarose gel. The PCRs on RNA and NTCs do not show products. PCR products of gDNA have expected sizes of 187 bp (sr10767 and sr10767ΔSP), 226 bp (sr16441 and sr16441ΔSP), 283 bp (sr14220), 237 bp (sr14226), and 210 bp (sr10529). PCRs on cDNA have expected sizes of 187 bp (sr10767 and sr10767ΔSP), 226 bp (sr16441 and sr16441ΔSP), 156 bp (sr14220), 168 bp (sr14226), and 128 bp (sr10529). The samples of the upper and lower panels were run on the same gel, respectively, photographed, and the picture was cut and reassembled for easier labeling of the lanes.

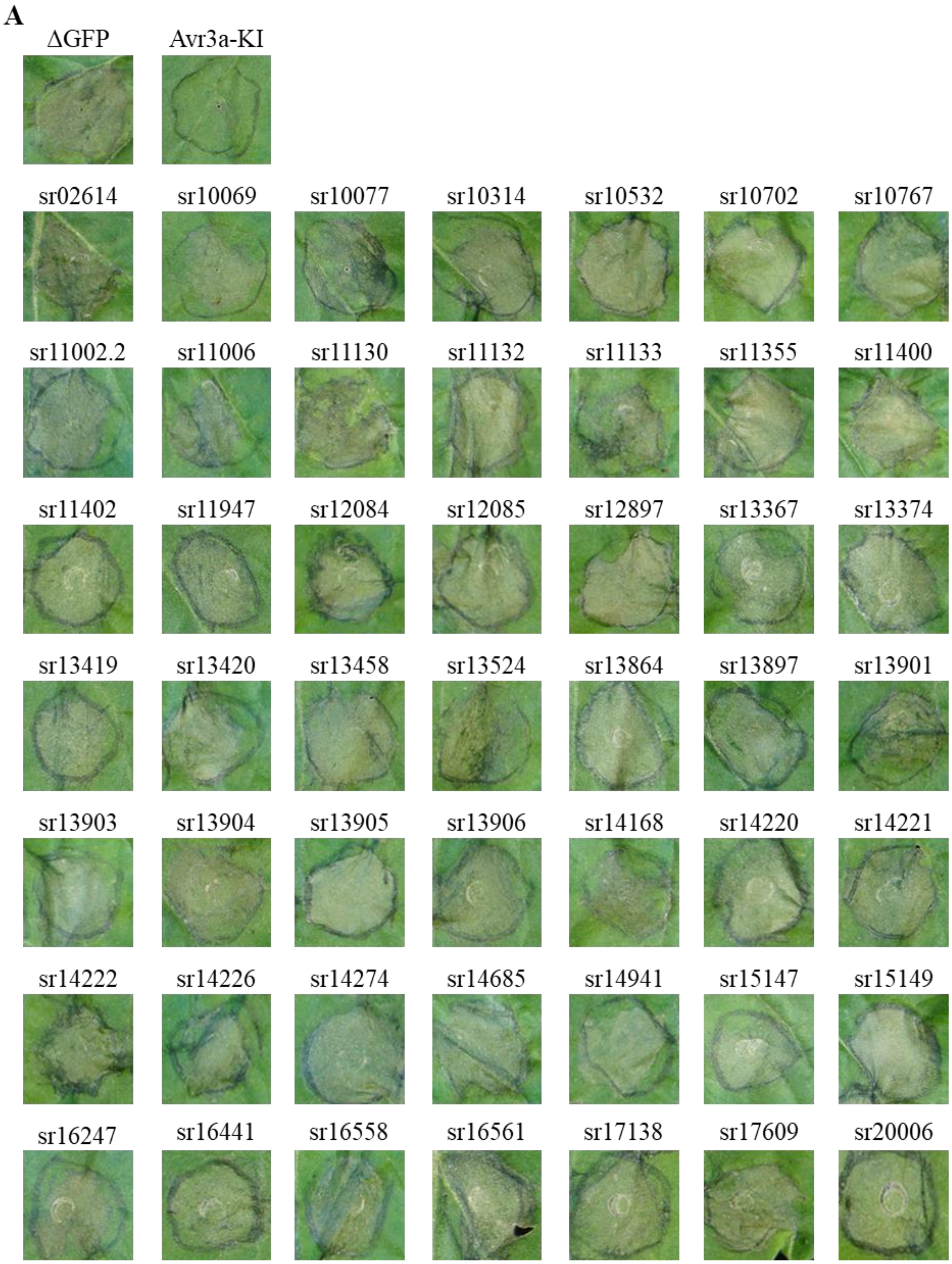
A
ΔGFP
Avr3a-KI
sr02614
sr10069
sr10077
sr10314
sr10532
sr10702
sr10767
sr11002.2
sr11006
sr11130
sr11132
sr11133
sr11355
sr11400
sr11402
sr11947
sr12084
sr12085
sr12897
sr13367
sr13374
sr13419
sr13420
sr13458
sr13524
sr13864
sr13897
sr13901
sr13903
sr13904
sr13905
sr13906
sr14168
sr14220
sr14221
sr14222
sr14226
sr14274
sr14685
sr14941
sr15147
sr15149
sr16247
sr16441
sr16558
sr16561
sr17138
sr17609
sr20006

B

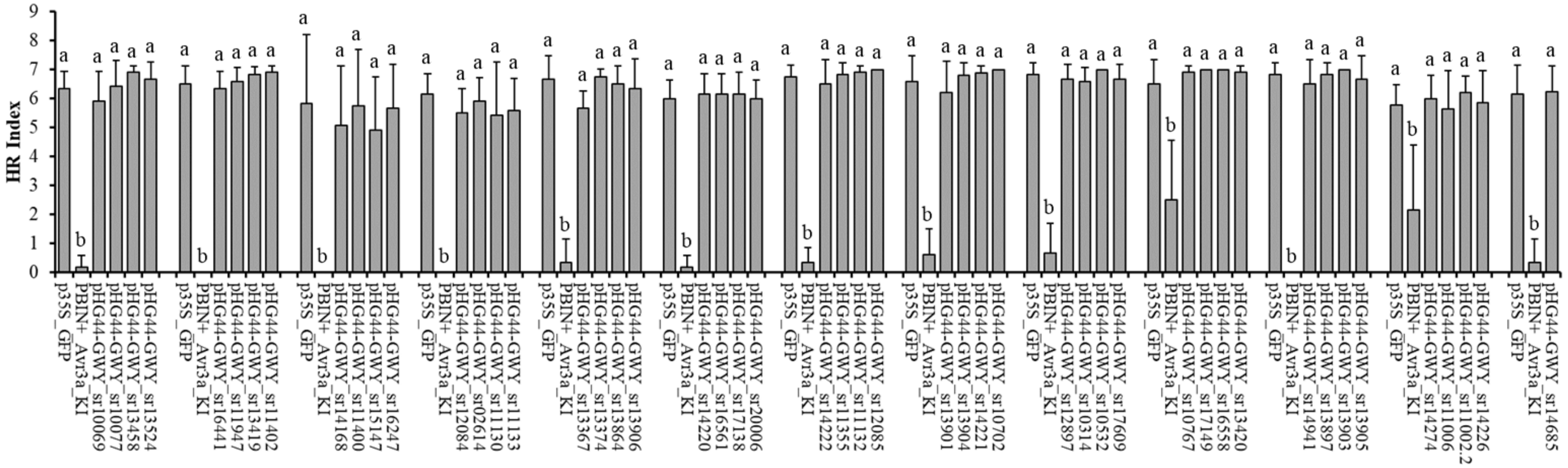

Figure S3. PCD suppression assay. The assay was conducted at TSL, UK. Agrobacterium strains carrying constructs with SRZ effectors were infiltrated in *N. benthamiana* along with GFP (negative control) and Avr3a-KI (positive control), one day later the infiltration sites were challenged with the elicitin INF1. The evaluation was done from 3-5 days after infiltration. (A) None of the 49 constructs tested in this experiment could suppress INF1-induced cell death (B) Quantitative comparison of necrosis induced by INF1 in the presence of PCD suppression candidates show no PCD suppression by SRZ effectors. The columns shows the mean and standard deviation. The letters above each column indicate statistically significant differences of the HR index ($P < 0.01$).
